# Funding free and universal access to Journal of Neuroinflammation

**DOI:** 10.1186/1742-2094-1-19

**Published:** 2004-10-14

**Authors:** Robert E Mrak, W Sue T Griffin

**Affiliations:** 1 Department of Pathology, University of Arkansas for Medical Sciences, Little Rock, Arkansas, USA; 2 Department of Geriatrics, University of Arkansas for Medical Sciences, Little Rock, Arkansas, USA

## Abstract

*Journal of Neuroinflammation *is an Open Access, online journal published by BioMed Central. Open Access publishing provides instant and universal availability of published work to any potential reader, worldwide, completely free of subscriptions, passwords, and charges. Further, authors retain copyright for their work, facilitating its dissemination. Open Access publishing is made possible by article-processing charges assessed "on the front end" to authors, their institutions, or their funding agencies. Beginning November 1, 2004, the *Journal of Neuroinflammation *will introduce article-processing charges of around US$525 for accepted articles. This charge will be waived for authors from institutions that are BioMed Central members, and in additional cases for reasons of genuine financial hardship. These article-processing charges pay for an electronic submission process that facilitates efficient and thorough peer review, for publication costs involved in providing the article freely and universally accessible in various formats online, and for the processes required for the article's inclusion in PubMed and its archiving in PubMed Central, e-Depot, Potsdam and INIST. There is no remuneration of any kind provided to the Editors-in-Chief, to any members of the Editorial Board, or to peer reviewers; all of whose work is entirely voluntary. Our article-processing charge is less than charges frequently levied by traditional journals: the *Journal of Neuroinflammation *does not levy any additional page or color charges on top of this fee, and there are no reprint costs as publication-quality pdf files are provided, free, for distribution in lieu of reprints. Our article-processing charge will enable full, immediate, and continued Open Access for all work published in *Journal of Neuroinflammation*. The benefits from such Open Access will accrue to readers, through unrestricted access; to authors, through the widest possible dissemination of their work; and to science and society in general, through facilitation of information availability and scientific advancement.

## Introduction

*Journal of Neuroinflammation *is an Open Access, online journal that is published by BioMed Central, an independent publisher committed to Open Access for peer-reviewed biomedical research [1]. Among the many benefits of Open Access publishing are (1) instant and universal availability of published work to any potential reader, worldwide, completely free of subscriptions, passwords, and charges; and (2) copyright retention by the authors rather than the publisher. This and many other benefits led us to select Open Access publishing, and to select BioMed Central, for the *Journal of Neuroinflammation*. Open Access publishing is made possible by article-processing charges (APCs) assessed "on the front end" to authors, their institutions, or their funding agencies. The *Journal of Neuroinflammation *will introduce APCs of around US$525 per article for manuscripts submitted on or after November 1, 2004. This charge will be waived for all authors from institutions that are BioMed Central members, and in additional cases for reasons of genuine financial hardship.

## Problems with the traditional publishing model

Traditional journals generally do not charge authors for publication (although assessed page or color charges may easily exceed our APCs). Instead, article access is traditionally paid for by readers, either through subscriptions or through fees assessed for online viewing and downloading. Over the past decade, escalating journal subscriptions have resulted in cash-strapped libraries cancelling journal subscriptions [2], thus limiting the range of articles available to many readers and limiting the potential audience available to authors.

## The Open Access publishing model

The *Journal of Neuroinflammation's *Open Access policy changes the way in which articles are published. First, all articles become freely and universally accessible online, immediately upon acceptance, so an author's work can be read by anyone at no cost. Second, the article authors retain copyright for their work, and grant to anyone the right to reproduce and disseminate the article, provided that it is correctly cited and no errors are introduced [1]. Third, a copy of the full text of each article is permanently archived in several separate online repositories. *Journal of Neuroinflammation's *articles are permanently archived in PubMed Central [3], the US National Library of Medicine's full-text repository of life science literature, and also in repositories at the University of Potsdam [4] in Germany, at INIST [5] in France and in e-Depot [6], the National Library of the Netherlands' digital archive of all electronic publications.

## Benefits of Open Access publishing

Open Access has four broad benefits for science and the general public. First, published work is disseminated freely and instantly to the widest possible audience, without barriers to access. Authors are free to reproduce and distribute their work at will, for example by placing it on their institution's website. Open Access publication has been shown to actually increase article citations and impact because of this easier availability [7]. Second, availability of Open Access articles enhances literature searching [8], as information available to researchers is not limited by what their libraries can afford. Third, results of publicly funded research are accessible to all taxpayers and not just those with access to libraries with journal subscriptions. Such public accessibility would actually become a legal requirement in the USA if the proposed Public Access to Science Act is enacted into law [9]. Fourth, article access is not limited by the economic resources of a scientist's country or institution; resource-poor countries and institutions are able to access the same material as wealthier ones, subject only to the availability of internet access [10].

## *Journal of Neuroinflammation's *article-processing charges

Article-processing charges will allow continued Open Access to all article published in *Journal of Neuroinflammation*. Authors will be asked to pay around US$525 upon acceptance of their article for publication. Submitted articles that are not accepted will incur no charge. There will be no charges for authors from institutions that are institutional members of BioMed Central. Currently this includes NHS England and all universities in the UK, the US National Institutes of Health and 136 other institutions and universities in the USA, the World Health Organization, and almost 200 additional institutions in 37 other countries [11]. Potential authors who are not associated with these institutions can avoid article-processing charges by getting their institution to join this list of BioMed Central institutional members. The annual institutional membership fee covers APCs for all authors at that institution for that year. In addition, many funding agencies have recognized the importance of Open Access publishing and have specified that funds from their grants may be used directly to pay APCs [12]. Finally, APC waivers are available for cases of genuine financial hardship. These will be considered on a case-by-case basis by the Editors-in-Chief.

## What do article-processing charges pay for?

The APC pays for an electronic submission process that facilitates efficient and thorough peer review, for publication costs involved in providing the article freely and universally accessible in various formats online, and for the processes required for the article's inclusion in PubMed and its archiving in PubMed Central, e-Depot, Potsdam and INIST. There is no remuneration of any kind provided to the Editors-in-Chief, to any members of the editorial board, or to peer reviewers; all of whose work is entirely voluntary. Although some authors may consider US$525 expensive, it must be remembered that *Journal of Neuroinflammation *does not levy any additional page or color charges on top of this fee. Because we are an online-only journal, any number of color figures, photographs, and 'extra' pages can be included at no extra cost. Such color and page charges, as assessed by more traditional journals, can easily exceed our flat US$525 per-article APC. Another common expense with traditional journals is the purchase of reprints for distribution, and the cost of these reprints is also frequently greater than our APCs. The *Journal of Neuroinflammation *provides free, publication-quality pdf files for distribution, in lieu of reprints.

## Free access versus Open Access

Several traditional journals now offer free access to their articles online, but this is different from Open Access as defined by the Bethesda Statement [13]. First, this access may be delayed for 6–2 months after publication. Second, readers are not free to reproduce and/or disseminate the work because of restrictions imposed by publishers' copyright policies. Even these restrictive policies do not ensure continued free access; the *British Medical Journal*, for instance, recently announced that it cannot continue to provide free access to its website [14]. They are considering various sources of revenue, including APCs [15].

APC-funded Open Access is not unique to BioMed Central or to the *Journal of Neuroinflammation*. The USA-based Public Library of Science (PLoS) is a new, non-profit organization that, like BioMed Central, is dedicated to online, Open Access publishing. PLoS has started two new Open Access journals, with APCs of US$1500 for each accepted article [16]. PLoS has used television advertising to promote their new journals [9], providing a high profile that should raise awareness of Open Access publishing in general. This, in turn, should encourage researchers in all disciplines to understand and accept Open Access, and to accept APCs as an acceptable funding method.

## Conclusion

Article-processing charges will enable full, immediate, and continued Open Access for all work published in *Journal of Neuroinflammation*. The benefits from such Open Access will accrue to readers, through unrestricted access; to authors, through the widest possible dissemination of their work; and to science and society in general, through facilitation of information availability and scientific advancement. We ask for your support in this important movement by submitting your next article to *Journal of Neuroinflammation *or to another Open Access journal.

## Competing interests

At Journal of Neuroinflammation, the work of the Editors-in-Chief, the Editorial Board, and of all invited outside peer reviewers is entirely voluntary, without tangible remuneration of any kind. Our goal is publication of biomedical research of the highest quality, and our (intangible) rewards lie in the achievement of these goals. Decisions about manuscripts are based entirely on the quality of the work, and not on the ability of authors to pay article-processing charges.

## Abbreviations

APC = article-processing charge.
